# Pfizer COVID19 vaccine is not associated with acute cardiovascular events excluding myocarditis– a national self-controlled case series study

**DOI:** 10.1186/s13584-024-00609-9

**Published:** 2024-04-24

**Authors:** Lital Keinan Boker, Ronen Fluss, Rita Dichtiar, Alina Rosenberg, Maya Ben-Lassan, Amit Huppert

**Affiliations:** 1 Israel Center for Disease Control, Ministry of Health, Sheba Medical Center, Gertner bldg, 52621 Tel Hashomer, Ramat Gan, Israel; 2 School of Public Health, Faculty of Social Welfare and Health Sciences, University of Haifa, Haifa, Israel; 3 Gertner Institute for the Study of Epidemiology and Health Policy, Sheba Medical Center, Ramat Gan, Israel; 4 School of Public Health, Sackler Faculty of Medicine, Tel Aviv University, Tel Aviv, Israel

**Keywords:** Acute cardiovascular events, Israel, Pfizer COVID19 vaccine, Self-controlled case series

## Abstract

**Background:**

Despite publications assuring no increased risk for acute cardiovascular events (excluding myocarditis) and sudden death following administration of COVID19 vaccines, these issues still stir much public ado. We assessed the risk for acute cardiovascular events that require hospitalization (excluding myocarditis) and for mortality in the short-term following administration of the second dose of the Pfizer COVID19 vaccine in Israel.

**Methods:**

Using a self-controlled case series (SCCS) study design and national databases, all second-dose vaccinees, who had not been diagnosed with COVID19 and who had an acute cardiovascular event (acute myocardial infarction/acute stroke/acute thromboembolic event) that required hospitalization in the 60 days following vaccine administration between Jan 11th, 2021 and Oct 31st 2021, were included. A similar analysis was carried out for mortality. The first 30 days following vaccination were defined as risk period while the next 30 days were defined as control period. The probability for an event between these periods was compared using a conditional logistic regression model, accounting for sex, age group, background morbidity and seasonal risk.

**Results:**

Out of 5,700,112  second dose vaccinees, 4,163 had an acute cardiovascular event in the 60 days following vaccine administration. Following exclusion of 106 due to technical considerations, 1,979 events occurred during the risk period and 2,078 during the control period: Odds ratio, OR = 0.95, 95% confidence interval, CI 0.90–1.01, *p* = 0.12. Adjusted OR was similar (OR = 0.88, 95%CI 0.72–1.08). Stratifying by age showed no increased risk in any age group. Mortality assessment indicated low number of events in both periods. These results were consistent in sensitivity analyses.

**Conclusions:**

There was no increased risk for acute cardiovascular events (excluding myocarditis) in the risk period compared to the control period following administration of the second dose of Pfizer COVID19 vaccine. Mortality data raised no concerns either, but may have been biased.

**Supplementary Information:**

The online version contains supplementary material available at 10.1186/s13584-024-00609-9.

## Background

The COVID19 pandemic, which is ongoing since early 2020, has had a profound impact globally, affecting the lives of millions of people: by the beginning of August 2023, over 692 million people worldwide have been diagnosed with SARS-CoV-2 virus, and over 6.9 million have died of the disease [1]. Vaccines play a vital role in combating the spread of infectious disease and in reducing its mortality and morbidity burden [2], especially when a novel pathogen is introduced. The speedy development of safe and efficient COVID19 messenger-RNA vaccines [3, 4], was a game changer in the current pandemic [5]. In Israel, Pfizer-Biotech BNT162b2 mRNA Covid-19 vaccine was gradually distributed since December 2020; by July 1st, 2021, out of a population of 9.4 million residents, 5,652,147 (60.4%) and 5,204,214 (55.6%) Israeli individuals were vaccinated by first and second vaccine doses, respectively [6].

Waning of vaccine efficacy became evident in Israel by mid-2021, when the Delta variant wave peaked [7, 8]. This waning, combined with real-world reports of rare but serious adverse events of the Pfizer-Biotech vaccine, such as myocarditis [9–11], pandemic fatigue, and vivid anti-vaccine campaigns in the social media often including baseless information and fake news, stirred a lot of public ado and often resulted in vaccine hesitancy and refusal [12]. By Jan 1st, 2022, a total of 4,264,929 (45.5%) individuals [6] adopted the (globally unprecedented until that time point) Israeli recommendation for a booster dose issued by the end of July 2021 as means to control the Delta variant COVID19 wave [13]; but only 829,896 (8.8%) responded by June 30th, 2022 to a following national recommendation for a second booster dose [6], issued by the end of January 2022 in light of the fast spread of the Omicron variant.

While myocarditis has been proved to be a potential rare adverse effect of mRNA COVID19 vaccines mostly affecting young male adults [9–11], it was also generally agreed that its risk was outweighed by the risk for myocarditis imposed by SARS-CoV-2 infection [14]. Unfortunately, public confusion regarding COVID19 vaccines have been fueled by sporadic reports on potential association between mRNA COVID19 vaccines and acute cardiovascular events as well as sudden death [15, 16]. Publication of other well-designed, peer-reviewed reports from France, Hong-Kong, Australia, New Zealand and Israel addressing these outcomes (excluding myocarditis) and usually concluding that there is no evidence for such associations [17–22], did not resolve these worries.

In light of these circumstances, policymakers in the Israel Ministry of Health (MoH) raised concerns regarding public response to future recommendations on booster doses of bivalent or other COVID19 vaccines. The Israel Center for Disease Control, a research body in the Israel MoH, assessed the risk for acute cardiovascular events that require hospitalization (excluding myocarditis) and for death in the short-term following administration of the second dose of the Pfizer COVID19 vaccine in Israel, using national databases and a self-controlled case series (SCCS) study design, in order to enable MoH evidence-based policy recommendations re. COVID19 vaccination. The null hypothesis was that risk and control time periods did not substantially differ with respect to the outcome probability.

## Methods

This is a SCCS, based on the total Israeli population. This study design is currently well accepted for the research of vaccine adverse effects [23]. A benefit of a SCCS is that it treats individuals as their own control, thereby minimizing measured or unmeasured confounding by variables that are time-insensitive. Another advantage is the smaller number of patients needing data extraction (or collection) as compared to a cohort study, especially when rare outcomes are considered.

### Study participants

The study population included all Israeli individuals who were vaccinated by a second Pfizer mRNA COVID19 vaccine in the time period from Jan 11th, 2021 up to Oct 31st 2021, who were not identified as COVID19 positive cases before or during this time period, to avoid potential confounding by an acute infection. This timeframe was chosen as it spans from the date of the first 2nd vaccine dose administration in Israel, up to when data on PCR COVID19 tests became less complete.

In this study we have focused on 2nd and not on 1st dose vaccinees, because of the short time gap between the two doses (three weeks) and the fact that 92% of 1st dose vaccinees were vaccinated by a second dose immediately afterwards. In sensitivity tests we have also examined 3rd dose vaccinees but did not include them in the main analysis, since by Oct 31st, 2021 3,970,474 Israeli citizens– 69% of 2nd dose vaccinees– undertook a 3rd vaccine dose, and they may represent a selective group, as the first recipients of the booster dose were older adults.

Study follow-up for each participant included 60 days following administration of the 2nd vaccine dose.

To address the study aim concerning acute cardiovascular events (excluding myocarditis), we have included 2nd dose COVID19 Pfizer vaccinees who have not been diagnosed with COVID19 and who have been hospitalized with an acute cardiovascular event (excluding myocarditis) during the designated follow-up time. If more than one hospitalization due to the defined diagnoses occurred during follow up, we have considered the first hospitalization only. Individuals who were hospitalized due to these diagnoses 60 days or less before their 2nd vaccine dose administration date were excluded, to avoid incidence-prevalence bias. Individuals with hospitalizations due to the defined cardiovascular events in the last 10 years (excluding the 60 days preceding vaccine administration) were considered as having a history of cardiovascular morbidity, which was accounted for in multivariable analysis.

To address the study aim focusing on mortality, we have included 2nd COVID19 Pfizer vaccine vaccinees who have not been diagnosed with COVID19 and who have died during the follow-up time.

### Data sources and study procedure

In order to answer both study questions we have used four MoH national databases:

1. ***The national SARS-CoV-2 vaccine database*** includes the name, lot number and dose number of the vaccine administered and the date of administration for each individual.
2. ***The national SARS-CoV2 test database*** includes the results of each COVID19 test performed, date of testing, and date of results delivery for each individual. This database had been established at the beginning of the pandemic as part of a large-scale program of contact tracing and isolation with widespread RT-PCR testing among suspected cases and contacts of confirmed COVID19 patients. Up to the last quarter of 2021, this database mostly included results of PCR tests. Once antigen tests were available in Israel (mid-2021), supervised antigen tests were also included in the dataset, either obliging isolation (if positive) or granting entitlement to a Green Pass (if negative), which served as a strategy to ease limitations on international travel, school closures, and social and physical distancing. By the end of 2021, the number of unsupervised domestic antigen tests– which were not reported to the national database - increased substantially, and compromised the completeness of this database. Therefore, as mentioned earlier, our recruitment ended by 31st October 2021.
3. ***The National Hospital Discharge Database (NHDD).*** The NHDD in the MoH captures every hospitalization occurring in all general hospitals in Israel. Data are uploaded periodically by every medical facility and are usually complete and validated within 6–12 calendric months. The database contains encrypted unique personal identification number, admission number, admission and discharge dates, demographic data, and discharge diagnoses. International Classification of Disease, 9th Edition (ICD-9) diagnose codes applied to define acute cardiovascular events included acute myocardial infarction (MI) (410), stroke (433.01, 433.11, 433.21, 433.31, 433.81, 433.91, 434, 436, 437.0, 437.1) and thromboembolic event (286.6, 325, 415.1, 434, 435,436, 437.6, 437.8, 437.9, 443, 444, 445, 451, 452). Myocarditis codes (420.9, 422, 423.9, 429.0) were excluded from the main analyses but used for sensitivity tests.
4. ***The National Population Registry*** includes the vital status of each Israeli resident.

The unique personal identity number each Israeli citizen has enabled a cross linkage of these databases in order to assembly the study populations. First, we identified all Pfizer COVID19 2nd dose vaccinees in the designated study period in Israel, and excluded those with a positive PCR test at any time point up to Oct 31st, 2021. This primary dataset was linked with the NHDD (following encryption of the dataset in the same manner as that of the NHDD) and also with the National Population Registry, to address both study questions.

As mentioned earlier, all study participants, both in the acute cardiovascular event analysis and the mortality analysis, were followed up for 60 days following their 2nd vaccine dose administration. For the acute cardiovascular event study question, the risk period was re-defined as 2-30d following 2nd vaccine administration, since the number of hospitalizations on day 1 (the first day after vaccination) was very low and may have represented a selection bias. Concurrently, the control time period was re-defined as 31-59d following 2nd vaccine administration, omitting the last day in order to balance follow up lengths. For the mortality study question, the corresponding periods were defined as 1-30d and 31-60d, respectively.

### Data analysis

First, we have built an epidemiological curve, e.g., a histogram that describes the numbers of each outcome following vaccination date, by study periods (risk and control).

Sociodemographic characteristics of the study population are described by numbers and percent for categorical variables or by mean (standard deviation), median and interquartile range for continuous variables.

Estimating the increased risk for the outcome in the risk period relative to the control period was done by using a univariable logistic regression model conditioning on subject and a multivariable model accounting for the following potential moderators: sex, age group (0-29y, 30-59y, 60-79y, 80 + y), previous cardiovascular morbidity in the last decade, and the seasonal risk of each study period. This was done by obtaining the average incidence for each acute cardiovascular event (excluding myocarditis) by each calendric month in pre-pandemic 2019 from the NHHD and using it to calculate, per each vaccinee, the relative risk for such an event in the two study periods. This multivariable analysis was repeated, stratified by age groups.

Sensitivity analyses for the acute cardiovascular event study question included (i) excluding vaccinees with previous cardiovascular morbidity in the last 10 years; (ii) extending the follow up period to 100 days, defining the first 50 days following the 2nd dose administration date as risk period and the next 50 days as control period.; (iii) including myocarditis cases in the defined cardiovascular events; and (iv) assessing the risk for the outcome following the 3rd vaccine dose using a model similar to that in the main analysis. Sensitivity analysis for the mortality question included extending the follow up period to 180 days, defining the first 90 days following the 2nd dose administration date as risk period and the next 90 days as control period. We have also assessed the relative risk of death between the 2nd dose vaccinees in 2021 and the general Israeli population in pre-pandemic 2019. Last, we also examined the mortality histogram following the 3rd vaccine dose.

All analyses were done using the R software package [24].

## Results

The total 2nd dose Pfizer vaccine population with no documented COVID19 infection in the period between Jan 11th 2021 to Oct 31st 2021 included 5,700,112 individuals. Of them, 4,163 were hospitalized with acute cardiovascular event (excluding myocarditis) while 1,815 have died within 60 days of their vaccine date.

### Acute cardiovascular event analysis (myocarditis excluded)

Of the total of 4,163 hospitalizations due to acute cardiovascular events in days 0–60 following 2nd vaccine dose administration, 1,979 hospitalizations occurred during the risk period (days 2–30) while 2,078 occurred in the control period (31-59d). A total of 106 cases, occurring on days 0, 1 and 60, were excluded from the analyses.

**Fig. 1:**
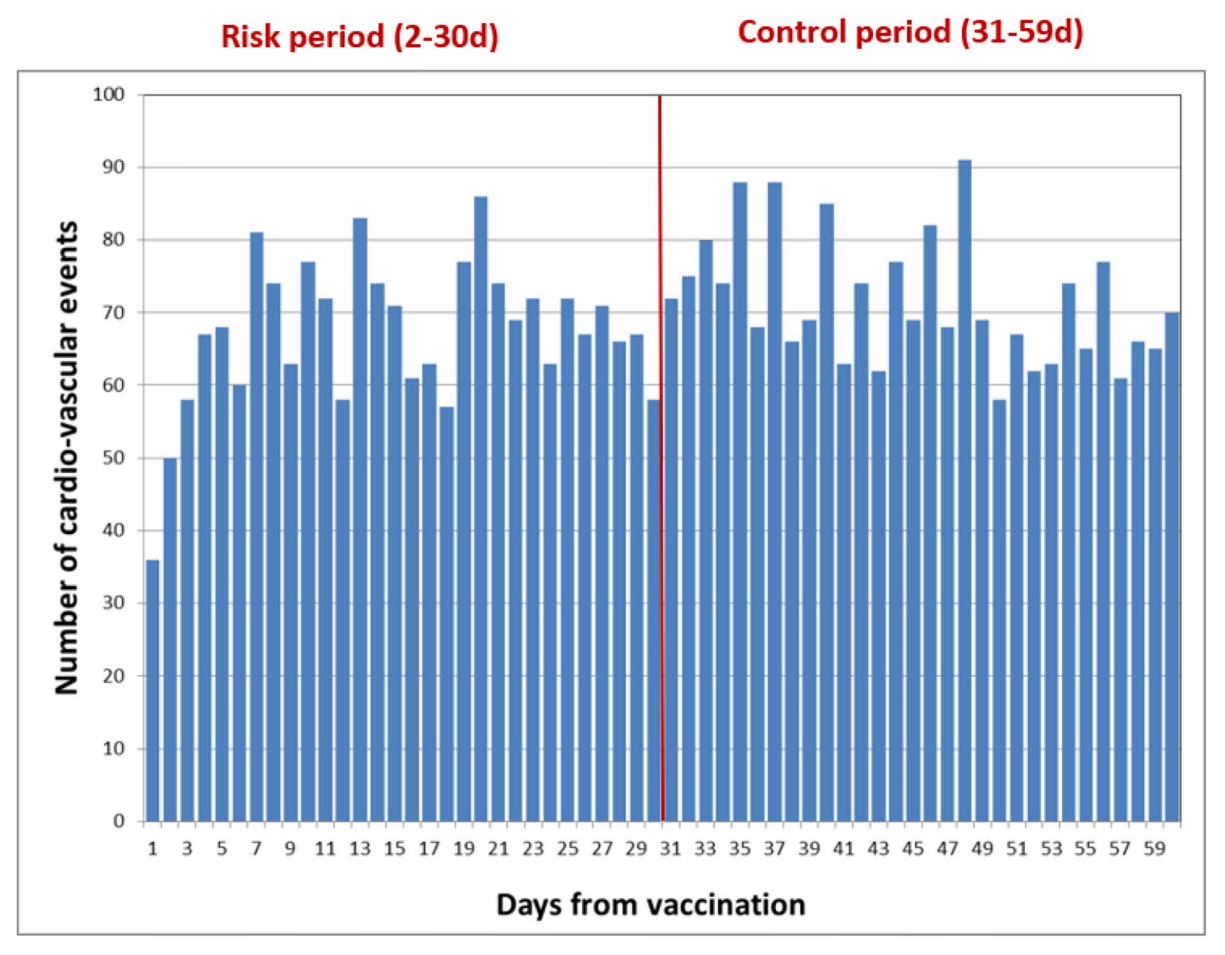
Distribution of acute cardiovascular hospitalizations (excluding myocarditis) following administration of the 2nd Pfizer COVID19 vaccine dose by study periods

Mean age of the total vaccinee group was 68.9y (standard deviation = 13.7y), median age was 70y (interquartile range = 60y,79y); 62% (*N* = 2,523) were males and 31% (*N* = 1,258) had hospitalizations due to acute cardiovascular morbidity (excluding myocarditis) in the last decade. No differences in age (68.7y vs. 69.0y, *p* = 0.41), sex (61.5% vs. 62.5% males, *p* = 0.69) or previous cardiovascular hospitalization (30% vs. 32%, *p* = 0.19) were observed between vaccinees whose events occurred in the risk period as compared to the control period, respectively. Likewise, the distributions of the different acute cardiovascular events were similar in both risk periods: acute MI accounted for 43% and for 42% of the hospitalizations in the risk and the control periods, respectively. The corresponding proportions for stroke/transient ischemic attack were 47% and 49%; for thromboembolic events the proportions were 7% and 6%, respectively, and for other diagnoses, 3% of all hospitalizations in both risk and control periods. Figure 1 shows the daily number of hospitalizations due to acute cardiovascular events (excluding myocarditis) by study period.

The non-adjusted conditional odds ratio (OR) was 0.95 and the 95% confidence interval (95%CI) was 0.90–1.01. The results of the conditional logistic regression model, which included the potential moderators: sex, age group, previous cardiovascular morbidity and seasonal risk are presented in Table 1. The OR for an acute cardiovascular event, myocarditis excluded, in the risk vs. the control period was 0.89 (95%CI 0.72–1.09) supporting the null hypothesis of similar risks in the two study periods.

**Table 1:** Odds ratios and 95%CI for an acute cardiovascular event (excluding myocarditis) by study periods– a multivariable conditional logistic regression model

Variable	Odds ratio (95%CI)	X^2^	Freedom levels	*P* value
Study period (risk vs. control)	0.88 (0.72–1.09)	3.9	1	0.48
Sex (females vs. males)	1.03 (0.90–1.18)	0.20	1	0.65
Age group		4.05	3	0.26
0-29y	1.79 (0.84–3.82)			
30-59y	1.12 (0.96–1.31)			
60-79y	1.00 (ref)			
80 + y	1.03 (0.88–1.20)			
Previous cardiovascular morbidity (yes vs. no)	0.93 (0.81–1.06)	1.08	1	0.30
Seasonal risk^1^	0.88 (0.72–1.07)	1.54	1	0.21

^1^Computed as the relative risk for an acute cardiovascular event causing hospitalization for each participant at their designated risk vs. control period, based on the 2019 NHDD data

Table 2 presents results of uni-and multivariable analyses, stratified by age groups. The only statistically significant result was a decreased OR in the age group 60–79 in the univariable analysis (OR = 0.91, 95%CI 0.84–0.99).

**Table 2:** Odds ratios and 95%CI for an acute cardiovascular event (excluding myocarditis) by study periods (risk vs. control) stratified by age group–conditional logistic regression models

	Unadjusted model		Adjusted model^1^	
Age group	Odds ratio of study period (95%CI) [Risk vs. control]	*P* value	Odds ratio of study period (95%CI) [Risk vs. control]	*P* value
0-29y^2^	1.64 (0.77–3.46)	0.20		
30-59y	1.03 (0.90–1.17)	0.68	1.19 (0.79–1.79)	0.40
60-79y	**0.91 (0.84–0.99)**	**0.04**	0.88 (0.67–1.15)	0.34
80 + y	0.95 (0.83–1.07)	0.38	0.80 (0.52–1.25)	0.335

^1^Each line represents the results of an age-specific model, adjusted for sex, previous cardiovascular morbidity and seasonal risk

^2^Could not include covariates due to small number of cases (*N* = 29)

Bold font denotes statistical significance at *p* < 0.05

The sensitivity analyses which included only vaccinees with no previous cardiovascular morbidity in the last 10 years, and the one that extended the follow up period to 100 days, showed results that were similar to those of the main analyses (data not presented). The sensitivity analysis which included also myocarditis cases, showed a statistically significant increased risk in the younger age group (0-29y): unadjusted OR = 3.8, 95%CI 2.3–6.3. The results of the analysis focusing on acute cardiovascular events subsequent to the 3rd vaccine followed those of the main analysis: OR in univariable model = 0.96 (95%CI 0.89–1.03), *p*-value 0.28, and OR in multivariable model = 1.08 (95%CI 0.85–1.38), *p*-value 0.53.

### Mortality analysis

**Fig. 2:**
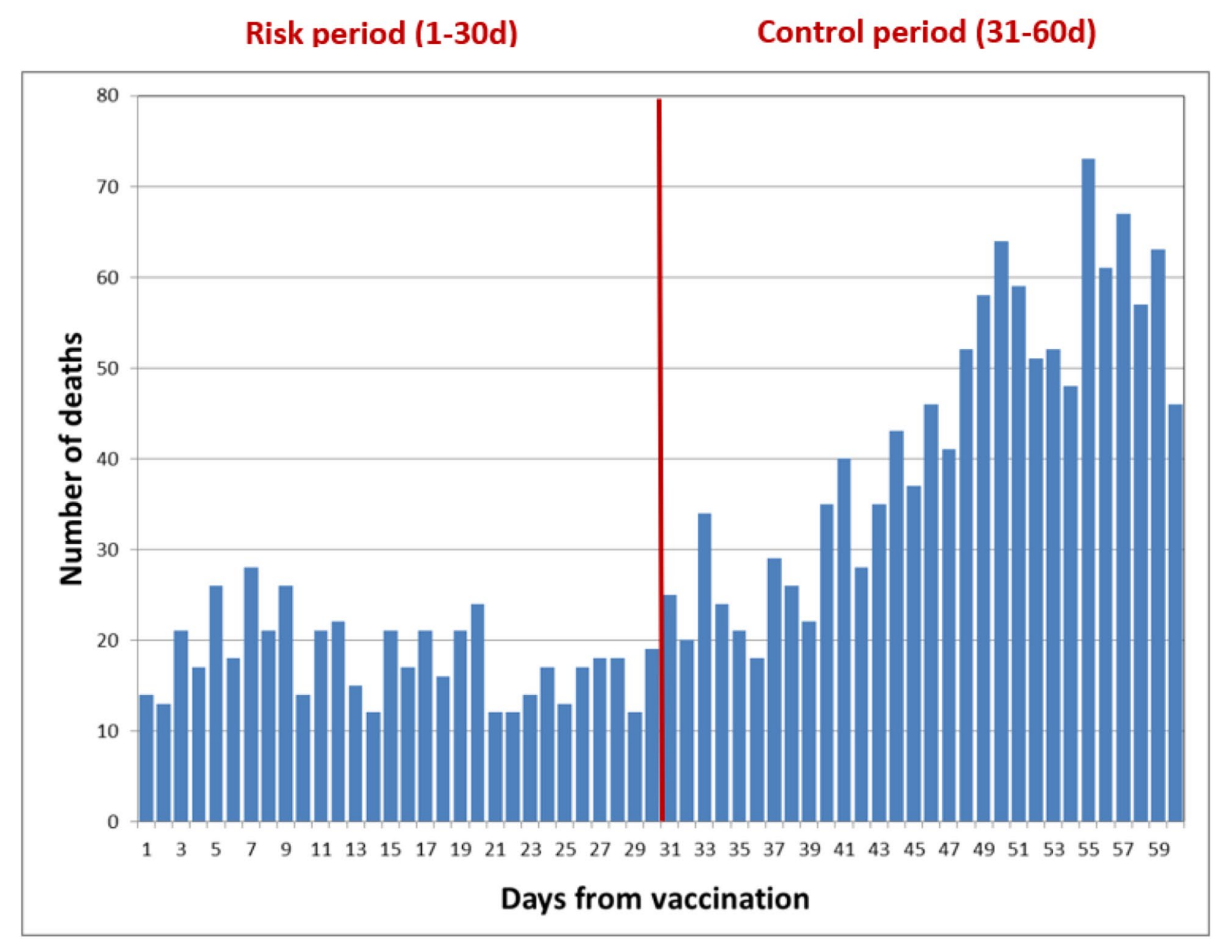
Distribution of mortality events following administration of the 2nd Pfizer COVID19 vaccine dose by study periods

Of the total of 1,815 death events observed in days 0–60 following 2nd vaccine dose administration, 540 occurred during the risk period (within 30 days of vaccine dose administration) while the rest (*N* = 1,275) occurred in the control period (31-60d following vaccine administration). Figure 2 shows the distribution of daily mortality since 2nd vaccine dose administration by study period. The striking differences between the two periods raised concerns regarding the validity of the results, thus we applied several sensitivity tests to clarify this concern. In Figure S1 we extended the follow-up to 180 days from the 2nd vaccination. Clearly, mortality increased steadily from the day of vaccination to about 50 days, after which the number was relatively stable and did not return to the low level seen immediately after vaccination. The peaks occurring after 50 days may be due to seasonality in the year as most people were vaccinated in January-February 2021.

As a further sensitivity check, we compared the risk of death among vaccinated subjects in the risk and the control periods (of 50 days each) with the average population mortality rate in 2019 (Table S1). In the risk period, the RR for the vaccinees was 0.25–0.35 whereas in the control period it was higher (0.55–0.66) but still less than 1.

Last, we looked at a histogram for the 3rd vaccination (Figure S2), which showed a pattern similar to that of the 2nd vaccination, with an increase in the occurrence of death up to day 50 following vaccination, and stable numbers with occasional peaks afterwards.

Since our sensitivity tests were more supportive of the possibility of a bias than of other explanations, we did not analyze the mortality results further.

## Discussion

The current SCCS study, based on Israel national databases, examined whether risk for acute cardiovascular events (excluding myocarditis) and mortality within 30 days of administration of the 2nd Pfizer vaccine dose against COVID19, was higher than the risk during the next 30 days. The results indicated that there were no substantial differences with respect to acute cardiovascular events (excluding myocarditis), even after accounting for potential moderators. Mortality data raised no concerns either as the number of events was low in both study periods, but since a potential bias has not been ruled out, no firm conclusions could be drawn.

### Acute cardiovascular events (excluding myocarditis)

Our results with respect to acute cardiovascular events (excluding myocarditis) are in accordance with a recent meta-analysis which focused on cardiovascular safety of these vaccines in real-world studies [25]; the authors reported no increased risk for myocardial infarction (risk ratio, RR = 0.98, 95%CI 0.87–1.09) or arrhythmia (RR = 0.98, 95%CI 0.84–1.12) following COVID19 vaccination. They did disclose increased moyocarditis occurrence, especially following 2nd and 3rd vaccine doses and in mRNA vaccines [25]. Another retrospective cohort study (with over 220,000 participants) focused on thrombotic outcomes in COVID19 vaccinees, and its results supported the safety of mRNA vaccines with respect to such events [26].

Several former SCCS studies from different countries, using different outcome definitions and follow up periods, largely reported similar results. A French SCCS, focusing on pulmonary emboli, acute MI, hemorrhagic or ischemic stroke in COVID19 vaccinees aged < 75y, found no association with the administration of mRNA COVID19 vaccines during a 21d follow-up [17]. In New Zealand, a SCCS study design was used to assess the risk for different thromboembolic events following the administration of Pfizer COVID19 vaccine during a 21d follow-up. The researchers reported no risk differences, even when stratified by ethnic origin [21]. Furthermore, a SCCS study from Hong-Kong which focused on high-risk individuals with known cardiovascular disease and explored a composite outcome of acute MI, stroke, revascularization and cardiovascular death, reported no evidence of an increased risk of these events after vaccination with Pfizer COVID19 vaccine [19]. These conclusions were further consolidated by a recent Israeli SCCS study, based on the dataset of the Clalit healthcare fund which covers around 50% of the Israeli population [22]. The study population included individuals at risk for COVID19 complications (e.g., aged 60 years or older, pregnant women, residents in nursing homes, and people with particular medical conditions such as chronic lung disease, chronic renal failure, hypertension and chronic heart failure) and focused on booster doses of the Pfizer vaccine. Primary outcome measures were the cumulative number of non-COVID19 hospitalizations and the cumulative number of non-COVID19 cardiovascular hospitalizations (including, among others, thromboembolic events and stroke, acute MI, arrhythmias, cardiac failure and myocarditis). Follow-up periods were 7d, 14d, 28d and 42d following vaccination, and the analyses were stratified by vaccine dose (first or second monovalent booster or first bivalent booster). No signal was identified in any of these analyses, and the authors concluded that these results further reassure the safety of the vaccine in high-risk individuals [22].

Thus, most current evidence, including this study, support the null hypothesis of no substantial differences in the probability for acute cardiovascular events (excluding myocarditis) between the risk and the control periods following vaccine administration. Myocarditis is an exception, and this fact is also supported by our sensitivity analysis, which did indicate a signal for the younger age group when myocarditis was included as an additional outcome.

### Mortality

The question of potentially higher risk for all-cause mortality in the short-term following administration of the 2nd COVID19 Pfizer vaccine dose could not be fully settled in this study.

Our results indicated a much **lower** risk for mortality in the short-term following vaccination, which increased and stabilized in the control period. Such results may suggest (i) a potential bias; (ii) a dramatic decrease during the risk period, followed by returning to normality in the control period; (iii) normality during the risk period, followed by a dramatic increase in the control period. The pattern observed– i.e., an increase over approximately 2 months followed by stable numbers afterwards - is more consistent with a healthy vaccinee effect [27] than with the option of a dramatic change in mortality. Furthermore, if vaccination dramatically increased mortality in the longer run, then one would expect to see a relative risk of greater than 1 for the control period, which– based on our sensitivity analysis comparing mortality rates in 2021 and 2019– was not the case. Therefore, a bias cannot be ruled out. The healthy vaccinee effect (similar to the better-known healthy worker effect) occurs due to persons who are not feeling well failing to attend their vaccination appointments. This phenomenon was strengthened by the Israel MoH’s regulation that prevented sick people from receiving the vaccination. The fact that the sensitivity analysis referring to 3rd dose vaccinees showed a similar pattern, further support this assumption. In fact, the healthy vaccinee effect is now well documented in the literature. For example, Høeg et al. [28] in their discussion, warn of the bias arising from the use of the newly vaccinated as controls, due to this effect. In our study, this effect continued up to 50 days post vaccination.

Two former publications implied that COVID19 vaccines may be associated with higher risk for cardiac arrest [15] or cardiovascular mortality [16], fueling public concerns through scientific, popular and social media. The 2022 ecological study by Sun et al. [15] relied on a 2019–2021 dataset from the Israel National Emergency Medical Services and disclosed an increase of over 25% in calls due to cardiac arrest and acute coronary syndrome in 16–39y old subjects during January–May 2021, compared with the years 2019–2020. The authors reported that weekly emergency call counts were significantly associated with the rates of the 1st and 2nd vaccine dose administered to this age group but not with COVID19 infection rates; however, these associations were purely ecological. Furthermore, the validity of the recorded main reason for these calls is unknown, and the study dataset excluded other emergency services in Israel [15]. It should also be mentioned, that no increase in sudden cardiac death in the young age group was noted during this time period in Israel, based on national data. Sun et al.‘s [15] results were not replicated by another ecological study which relied on national databases and looked at out-of-hospital cardiac arrest (OHCA) in young people (1-50y) in Victoria, Australia [20]. The main results did not demonstrate increased rates of overall OHCA, myocarditis causing OHCA, or unascertained OHCA during the pandemic or after the introduction of COVID19 vaccination. Furthermore, causes of death in young people experiencing fatal OHCA within 30d of their COVID19 vaccination (any dose) were consistent with pre-pandemic causative profiles for this age group [20].

The SCCS report published by the Florida Health Department [16] indicated no differences in all-cause mortality in the first 28d following vaccination as compared to the control period, but rather a statistically significant mortality reduction in participants aged 60 + y (relative incidence = 0.97, 95%CI 0.94–0.99). On the other hand, cardiac-related mortality risk was significantly higher during the risk period for males (relative incidence = 1.09, 95%CI = 1.03–1.15) but not for females, and this effect was mostly seen for mRNA vaccines (male relative incidence = 1.11, 95% CI = 1.05–1.18) [16]. However, this report was not published in a peer-reviewed journal. A recent meta-analysis used three different SCCS publications on all-cause and cardiac-related mortality following COVID19 vaccination [29]. While the pooled hazard ratio (HR) revealed no significant association of COVID19 vaccination with all-cause mortality (HR = 0.89, 95%CI 0.71–1.10), the pooled HR for cardiac-related mortality was significantly increased (HR = 1.06, 95%CI 1.02–1.11), mainly in males [29]. Interestingly, these results were mostly driven by the Florida’s Health Department report [16], since the other two SCCS studies included in this meta-analysis [30, 31] showed no increase in mortality risk in the short-term period following COVID19 vaccine administration; the Italian SCCS study [30] focused on mRNA vaccines only and showed no increase for all-cause mortality on days 3,7,14 and 30 following vaccine administration [30] while the British SCCS study [31] focused on several types of COVID19 vaccines, and disclosed no significant increase in all-cause or cardiac mortality in the 12 weeks following COVID19 vaccination [31]. Thus, despite our inability to draw clear conclusions on mortality following Pfizer mRNA COVID19 2nd vaccine dose, the current scientific evidence mostly supports no such association, and our results raise no concerns regarding increased mortality in the shorter and longer-term following vaccine administration.

Our study has several limitations. First, the follow up period spanned only 60 days following vaccine administration thus potential effects that may have occurred later than that were not captured. However, adverse effects of vaccines are usually expected to present earlier, not later, and our sensitivity analyses, which extended the follow up periods on both outcomes, did not show postponed effects. Second, we focused on the 2nd vaccine dose only. However, assessment of 3rd dose vaccinees in sensitivity tests showed very similar results. Another limitation of the current study is that we only had data on acute cardiovascular events which caused hospitalizations. Having said that, it should be kept in mind that most serious effects do require hospitalization. In addition, our outcome data were based on hospital ICD-9 diagnoses codes, without further verification in patients’ files. Nevertheless, since this methodology was equally used in both study periods, potential misclassifications are not expected to be differential. Furthermore, we introduced only few potential moderators into our models due to lack of information on others. However, the inherent SCCS study design reduces confounding by time-insensitive covariates. Additionally, we cannot rule out potential biases. Firstly, there is an inherent bias due to death being a one-time event. While an acute cardiovascular event may occur prior to the designated follow-up time, and also in both the risk and the control periods, death occurs only once and this occurrence prevents any other. However, since death is a relatively rare event, we do not expect this bias to be large. On the other hand, a potential healthy vaccinee effect, indicating a lower mortality rate among vaccinees vs. non-vaccinees for reasons other than the administration of the vaccine itself, may be more relevant and cannot be ruled out [27, 28]. Last, we had no information of specific death causes, since the relevant data for the year 2021 were not available in Israel at the time of the study.

On the other hand, the study has some advantages too. A strength of the study is it analyzes short-term cardiovascular events and mortality data in a controlled manner, based on the total population in Israel and on national validated databases. Its design accounted for potential confounding by time-insensitive individual covariates, such as comorbidities, and we were able to include moderators such as former cardiovascular hospitalizations and seasonal risk in our models. Last, we were able to exclude COVID19 positive individuals, thus reducing potential confounding by acute infection and by Long-COVID19 status.

## Conclusions

In this SCCS study, based on national databases, we found no differences in risk for an acute cardiovascular event (excluding myocarditis) in the early (2-30d) compared to the later (31-59d) time period following administration of the 2nd dose of the Pfizer COVID19 vaccine. Mortality data raised no concerns either, but may have been prone to bias. The findings of this study, undertaken by an applicative research unit in the Israel MoH in order to support policy recommendations, complete the information provided by a former Israeli study focusing on booster vaccine doses [22], and both studies further assure the safety of this vaccine. Subsequently, the current Israeli MoH recommendations are in favor of COVID19 booster vaccination.

### Electronic Supplementary Material

Below is the link to the electronic supplementary material.

Supplementary Material 1

## Data Availability

The national databases used in this study are available through the TIMNA (Hebrew acronym for “Research infrastructure for big data studies”) platform of the Ministry of Health. See: https://govextra.gov.il/ministry-of-health/big-data-research/home/.

## List of Abbreviations

CI: Confidence Interval
HR: Hazard Ratio
SCCS: Self-Controlled Case Series
MI: Myocardial Infarction
MoH: Ministry of Health
NHDD: The National Hospital Discharge Database
OHCA: Out-of-Hospital Cardiac Arrest
OR: Odds Ratio
RR: Risk Ratio

## References

[1] Worldmeter coronavirus website. https://www.worldometers.info/coronavirus/worldwide-graphs/. Accessed 31st August, 2023.

[2] Greenwood B (2014). The contribution of vaccination to global health: past, present and future. Philos Trans R Soc Lond B Biol Sci.

[3] Polack FP, Thomas SJ, Kitchin N, Absalon J, Gurtman A, Lockhart S, Perez JL, Pérez Marc G, Moreira ED, Zerbini C, Bailey R, Swanson KA, Roychoudhury S, Koury K, Li P, Kalina WV, Cooper D, Frenck RW, Hammitt LL, Türeci Ö, Nell H, Schaefer A, Ünal S, Tresnan DB, Mather S, Dormitzer PR, Şahin U, Jansen KU, Gruber WC, C4591001 Clinical Trial Group (2020). Safety and Efficacy of the BNT162b2 mRNA Covid-19 vaccine. N Engl J Med.

[4] Baden LR, El Sahly HM, Essink B, Kotloff K, Frey S, Novak R, Diemert D, Spector SA, Rouphael N, Creech CB, McGettigan J, Khetan S, Segall N, Solis J, Brosz A, Fierro C, Schwartz H, Neuzil K, Corey L, Gilbert P, Janes H, Follmann D, Marovich M, Mascola J, Polakowski L, Ledgerwood J, Graham BS, Bennett H, Pajon R, Knightly C, Leav B, Deng W, Zhou H, Han S, Ivarsson M, Miller J, Zaks T, COVE Study Group (2021). Efficacy and safety of the mRNA-1273 SARS-CoV-2 vaccine. N Engl J Med.

[5] Watson OJ, Barnsley G, Toor J, Hogan AB, Winskill P, Ghani AC (2022). Global impact of the first year of COVID-19 vaccination: a mathematical modelling study. Lancet Infect Dis.

[6] Israel Data World website. https://datadashboard.health.gov.il/portal/dashboard/corona. Accessed 31st August 2023.

[7] Levin EG, Lustig Y, Cohen C, Fluss R, Indenbaum V, Amit S, Doolman R, Asraf K, Mendelson E, Ziv A, Rubin C, Freedman L, Kreiss Y, Regev-Yochay G (2021). Waning Immune Humoral response to BNT162b2 Covid-19 vaccine over 6 months. N Engl J Med.

[8] Goldberg Y, Mandel M, Bar-On YM, Bodenheimer O, Freedman L, Haas EJ, Milo R, Alroy-Preis S, Ash N, Huppert A (2021). Waning immunity after the BNT162b2 vaccine in Israel. N Engl J Med.

[9] Mevorach D, Anis E, Cedar N, Bromberg M, Haas EJ, Nadir E, Olsha-Castell S, Arad D, Hasin T, Levi N, Asleh R, Amir O, Meir K, Cohen D, Dichtiar R, Novick D, Hershkovitz Y, Dagan R, Leitersdorf I, Ben-Ami R, Miskin I, Saliba W, Muhsen K, Levi Y, Green MS, Keinan-Boker L, Alroy-Preis S (2021). Myocarditis after BNT162b2 mRNA vaccine against Covid-19 in Israel. N Engl J Med.

[10] Mevorach D, Anis E, Cedar N, Hasin T, Bromberg M, Goldberg L, Parnasa E, Dichtiar R, Hershkovitz Y, Ash N, Green MS, Keinan-Boker L, Alroy-Preis S (2022). Myocarditis after BNT162b2 vaccination in Israeli adolescents. N Engl J Med.

[11] Mevorach D, Anis E, Cedar N, Hasin T, Bromberg M, Goldberg L, Levi N, Perzon O, Magadle N, Barhoum B, Parnassa E, Dichtiar R, Hershkovitz Y, Green MS, Ash N, Keinan-Boker L, Alroy-Preis S (2022). Myocarditis after BNT162b2 COVID-19 Third Booster Vaccine in Israel. Circulation.

[12] Frankenthal D, Zatlawi M, Karni-Efrati Z, Keinan-Boker L, Luxenburg O, Bromberg M (2022). COVID-19 vaccine hesitancy among Israeli adults before and after vaccines’ availability: a cross-sectional national survey. Vaccine.

[13] Ash N, Triki N, Waitzberg R (2023). The COVID-19 pandemic posed many dilemmas for policymakers, which sometimes resulted in unprecedented decision-making. Isr J Health Policy Res.

[14] Barda N, Dagan N, Ben-Shlomo Y, Kepten E, Waxman J, Ohana R, Hernán MA, Lipsitch M, Kohane I, Netzer D, Reis BY, Balicer RD (2021). Safety of the BNT162b2 mRNA Covid-19 vaccine in a nationwide setting. N Engl J Med.

[15] Sun CLF, Jaffe E, Levi R (2022). Increased emergency cardiovascular events among under-40 population in Israel during vaccine rollout and third COVID-19 wave. Sci Rep.

[16] Ladapo J. Exploring the relationship between all-cause and cardiac-related mortality following COVID-19 vaccination or infection in Florida residents: a self-controlled case series study. https://floridahealthcovid19.gov/wp-content/uploads/2022/10/20221007-guidance-mrna-covid19-vaccines-analysis.pdf [accessed Aug 2023].

[17] Botton J, Jabagi MJ, Bertrand M, Baricault B, Drouin J, Le Vu S, Weill A, Farrington P, Zureik M, Dray-Spira R (2022). Risk for myocardial infarction, stroke, and Pulmonary Embolism following COVID-19 vaccines in adults younger Than 75 years in France. Ann Intern Med.

[18] Wan EYF, Wang Y, Chui CSL, Mok AHY, Xu W, Yan VKC, Lai FTT, Li X, Wong CKH, Chan EWY, Lau KK, Cowling BJ, Hung IFN, Wong ICK (2022). Safety of an inactivated, whole-virion COVID-19 vaccine (CoronaVac) in people aged 60 years or older in Hong Kong: a modified self-controlled case series. Lancet Healthy Longev.

[19] Ye X, Ma T, Blais JE, Yan VKC, Kang W, Chui CSL, Lai FTT, Li X, Wan EYF, Wong CKH, Tse HF, Siu CW, Wong ICK, Chan EW (2022). Association between BNT162b2 or CoronaVac COVID-19 vaccines and major adverse cardiovascular events among individuals with cardiovascular disease. Cardiovasc Res.

[20] Paratz ED, Nehme Z, Stub D, La Gerche A (2023). No Association between Out-of-hospital cardiac arrest and COVID-19 vaccination. Circulation.

[21] Walton M, Tomkies R, Teunissen T, Lumley T, Hanlon T (2023). Thrombotic events following the BNT162b2 mRNA COVID-19 vaccine (Pfizer-BioNTech) in Aotearoa New Zealand: a self-controlled case series study. Thromb Res.

[22] Yamin D, Yechezkel M, Arbel R, Beckenstein T, Sergienko R, Duskin-Bitan H, Yaron S, Peretz A, Netzer D, Shmueli E. Safety of monovalent and bivalent BNT162b2 mRNA COVID-19 vaccine boosters in at-risk populations in Israel: a large-scale, retrospective, self-controlled case series study. Lancet Infect Dis. 2023 Jun 20:S1473-3099(23)00207-4. 10.1016/S1473-3099(23)00207-4 37352878

[23] Whitaker HJ, Hocine MN, Farrington CP (2009). The methodology of self-controlled case series studies. Stat Methods Medic Res.

[24] R Core Team. R: A language and environment for statistical computing. R Foundation for Statistical Computing, Vienna, Austria. 2019. URL https://www.R-project.org/

[25] Chang Y, Lv G, Liu C, Huang E, Luo B. Cardiovascular safety of COVID-19 vaccines in real-world studies: a systematic review and meta-analysis. Expert Rev Vaccines. 2023 Jan-Dec;22(1):25–34. 10.1080/14760584.2023.2150169 36413786

[26] Patterson WM, Greene BD, Tefera L, Bena J, Milinovich A, Mehta N, Chung MK, Kapadia S, Svensson LG, Cameron SJ (2023). Thrombotic outcomes in patients in a large clinical enterprise following COVID-19 vaccination. J Thromb Thrombolysis.

[27] Hitchings MDT, Lewnard JA, Dean NE, Ko AI, Ranzani OT, Andrews JR, Cummings DAT (2022). Use of recently vaccinated individuals to Detect Bias in Test-negative case-control studies of COVID-19 vaccine effectiveness. Epidemiology.

[28] Høeg TB, Duriseti R, Prasad V (2023). Potential healthy Vaccinee Bias in a study of BNT162b2 vaccine against Covid-19. N Engl J Med.

[29] Marchand G, Masoudi AT, Medi S (2023). Risk of all-cause and cardiac-related mortality after vaccination against COVID-19: a meta-analysis of self-controlled case series studies. Hum Vaccin Immunotherapeut.

[30] Stivanello E, Beghelli C, Cardoni F, Giansante C, Marzaroli P, Musti MA, Perlangeli V, Todeschini R, Pandolfi P (2022). Short-term mortality following COVID-19 vaccination in Bologna, Italy: a one-year study. Vaccine.

[31] Marxhand G, Nasoud AT, Medi S, Nafilyan V, Bermingham CR, Ward IL, Morgan J, Zaccardi F, Khunti K, Stanborough J, Banerjee A, Doidge JC (2023). Risk of death following COVID-19 vaccination or positive SARS-CoV-2 test in young people in England. Nat Commun.

