# Supplementary material for: Pfizer COVID19 vaccine is not associated with acute cardiovascular events excluding myocarditis– a national self-controlled case series study
Source: Isr J Health Policy Res. 2024 Apr 24;13:23. doi: 10.1186/s13584-024-00609-9 (PMC11040923; doi:10.1186/s13584-024-00609-9)

SUPPLEMENTS

Table S1: **Sensitivity analysis - relative risk (RR) of death between 2^nd^ dose Pfizer COVID19 vaccinees on 2021 and the general Israeli population on 2019**

| **Age Group** | **Risk period (1-50d)** | **Control period (51-100d)** |
| --- | --- | --- |
| **25-59** | 0.35 | 0.66 |
| **60-79** | 0.29 | 0.67 |
| **80+** | 0.25 | 0.55 |

Figure S1: **Sensitivity analysis - distribution of mortality events following administration of the 2^nd^ Pfizer COVID19 vaccine dose, with 180d follow up**


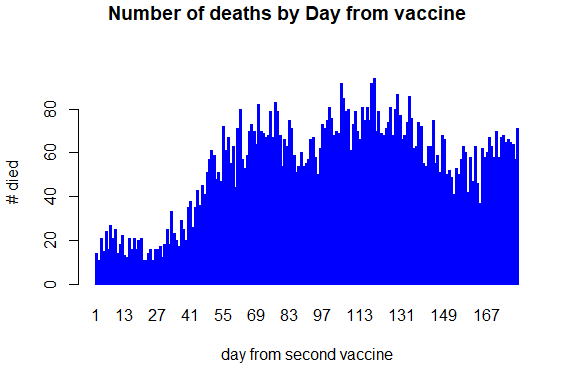


Figure S2: **Sensitivity analysis - distribution of mortality events following administration of the 3^rd^ Pfizer COVID19 vaccine dose, with 100d follow up**


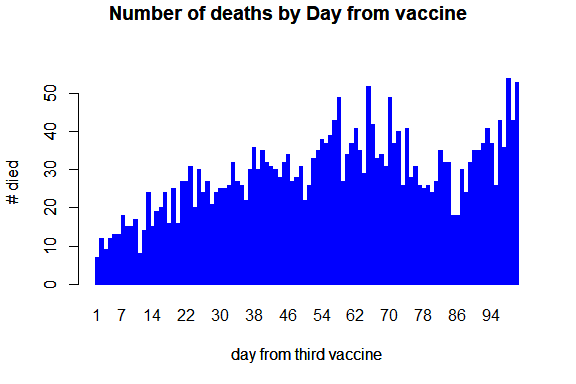

Supplement: Supplementary file 1 — Supplementary Material 1 [file 13584_2024_609_MOESM1_ESM.docx]
